# Alkali Activation of Milled Red Brick Waste and Calcined Illite Clay with Silica Gel Addition

**DOI:** 10.3390/ma15093195

**Published:** 2022-04-28

**Authors:** Girts Bumanis, Danutė Vaičiukynienė

**Affiliations:** Faculty of Civil Engineering and Architecture, Kaunas University of Technology, Studentų St. 48, LT-51367 Kaunas, Lithuania; danute.vaiciukyniene@ktu.lt

**Keywords:** geopolymers, illite clay, fineness, silica gel, strength, XRD, microstructure

## Abstract

The role of precursor characteristics and mixture composition design of alkali-activated materials (AAM) has been intensively researched with different types of alumino-silicate sources. Two illite-based precursors were prepared and investigated—(i) raw illite clay (IC) treated in a laboratory at 700, 750, and 800 °C and (ii) a red brick waste coming from the brick production plant. The fineness of precursors was determined and compared. The precursors were activated with 6 M and 7 M NaOH alkali solutions. Silica gel addition was considered in the composition of AAM. The XRD results indicate the transformation of both precursor types under alkali activation. The efflorescence salts were analyzed on the samples with silica gel addition. Calcined IC precursor allowed us to obtain AAM with a strength from 11 to 16 MPa with an increasing strength gain during curing. The red brick waste precursor showed a compressive strength from 14 to 28 MPa. A high early strength was obtained with no further strength increase. The hydrosodalite and zeolite crystals were detected in the structure of AAM based on the red brick waste precursor. The results indicate different characteristics of AAM based on similar source precursors, showing the important role of the proper treatment of precursors before alkali activation.

## 1. Introduction

Kaolinite is one of the main alumosilicate sources for alkali-activated material (AAM) development. Under temperature treatments between 600 and 900 °C, an amorphous structure of aluminum and silicon oxides is formed. The use of natural mineral earth components (particularly metakaolin) is now largely out of the question. Alternative and sustainable precursors have already been researched. The use of by-products as secondary materials such as fly ash and slags to form AAM has been widely researched while other alumosilicate sources are still pursued [1,2]. The main clays used in the preparation of AAM are 1:1 layer lattice alumosilicates, while the limited availability or lack of raw materials is bringing interest into 2:1 clay minerals, e.g., pyrophyllite, illite-smectite clays, etc. [3]. After heat treatment at 700 to 900 °C, clays rich in kaolinite are more reactive to alkali activation than clays dominated by smectite or illite [4]. These other clay minerals are commonly available around the world and may exhibit certain reactivity after heat activation. [5]. Alkali activation with a potassium and sodium hydroxide of 2:1 layer illite clay (IC) has been studied before, but thermal treatment was performed after raw clay activation with 6 M NaOH alkali activation. Afterward, thermal treatment between 600 and 900 °C led to the production of ceramic samples with a strength from 6 to 24 MPa [3]. Previous research has tested thermally treated IC between 550 and 950 °C and it was determined that the highest release of aluminate was reached at 750 °C [5]. In addition, montmorillonite and illite are both 2:1 clay minerals, however, their alkali activation behavior is very different and montmorillonite-rich soils proved to be more promising [6]. Si/Al ratios from 2.81 to 3.85 may lead to AAM with a strength of 7 to 26 MPa [7]. Since the presence of 2:1 clay minerals brings a high Si/Al ratio (>2), this brings limitations to the use of waterglass addition in the alkaline activator solution [5,8]. There are also reports where alkali solution with sodium silicate addition proved to result in low chemical stability of the AAM [9]. Dietel et al. concluded that the specific surface area of heat-treated IC is even more important than the amount of dissolved Si and Al, and Si/Al ratio in the geopolymerization [10]. Vasić et al. lately activated a mixture of calcined IC clay and red brick mixture with 10 M KOH and sodium silicate solution, which resulted in AAM with a strength up to 13.7 MPa [11]. Kaolinitic-illitic raw clay previously was activated with 4 to 14 M NaOH solution which resulted in a conclusion that the increase in curing temperature and NaOH concentration had a synergistic effect [12]. Good results were obtained by illite-smectite-rich clay activated with NaOH solution at 75 °C, which formed geopolymers with a strength of around 30 MPa [13]. This brings a promising perspective for IC use as an AAM precursor. The waste type precursor from IC would be more beneficial than heat treatment of raw clay at an elevated temperature.

Silica gel is a type of waste coming from the fertilizer production industry with limited application. To produce 1 t of AlF_3_, around 0.5 t of silica gel is obtained [14]. Silica gel is characterized by high amorphous phase content, mainly containing around 70% SiO_2_ and a smaller amount of Al_2_O_3_. High fluoride impurity content is characteristic of silica gel—it can be around 20% of the content forming aluminum fluoride hydrate (AlF_3_·3.5H_2_O), thus it partially reduces the content of free Al_2_O_3_ needed for the production of AAM. High fluorine content makes the use of silica gel use problematic and pyrolysis is one of the ways to remove AlF_3_ from the material [15]. The addition of silica gel has been used to prepare geopolymers in the range from 2 to 40%, while only up to 10% was the border where the mechanical performance of AAM was reduced drastically [14,16].

This research investigates the alkali activation efficiency of red brick waste resulting from the brick production process as a precursor for the production of AAM. For the first time, the fineness factor of red brick waste powder was investigated and compared to natural raw IC treated at 700 and 800 °C in a laboratory.

## 2. Materials and Methods

### 2.1. Raw Materials

A total of two clay treatment approaches were used to prepare precursors for the production of AAM samples. First, carbonate-free illite clay (IC) from the Liepa deposit in Latvia was prepared by thermal treatment. After the collection of clay samples from a clay deposit, samples were placed in a drying chamber at 105 °C until a constant mass was obtained. Dry clay was milled by collision milling using a semi-industrial disintegrator which is described in the previous paper [17]. At this stage, the differential thermal analysis / thermogravimetric analysis (DTA/TG) was performed. The raw IC powder was then heat-treated at 700, 750, and 800 °C for 4 h at a maximum temperature at a rate of 5 °C/min. For milled IC, the laser particle size distribution and X-ray diffraction (XRD) were determined. The other precursor originated from a brick production plant. Red brick waste was collected from the local brick production plant that is placed near the IC quarry (JSC Lode, Latvia). During the production, the bricks there are burned at a temperature between 900 and 950 °C. Then defective products are crushed and coarsely ground to brick sand at the factory. Red brick waste was ground similar to the raw IC. An additional grinding (3x more grinding energy applied) was done for red brick waste to reduce its particle size.

In the AAM composition, silica gel waste from a fertilizer manufacturing factory was included as a high silica additive. Silica gel was not additionally treated and was used with its naturally high moisture content (between 80 and 100 wt.%). The weight of the silica gel in the mixture composition was calculated according to its moisture content.

Commercially available sodium hydroxide flakes with 99% purity (Tianye Chemicals Ltd., Xinjiang, China) were used to prepare an alkali activation solution.

The chemical composition of raw IC, red brick waste, and silica gel are given in Table 1. Results show that red clay has 14.5% of Al_2_O_3_, and 70.3% of SiO_2_, which also corresponds to previous investigations and is an indication that an extra silica source from alkali activation solution is not needed [14,18]. A high amount of Fe_2_O_3_ is characteristic of ferric aluminosilicate clay deposits, which also gives an orange to red-brown color. Clay from the Lode deposit is characterized by a relatively high K_2_O content (4.0%) and MgO (1.1%) was also recorded. The red brick waste chemical composition indicates similar composition as for raw clay. According to XRF analysis, silica gel contains a high content of SiO_2_ (72.2%) and Al_2_O_3_ (5.68%). Such values give promising indications for using silica gel as a raw material in the composition of AAM. Minor values of CaO and Fe_2_O_3_ were also detected. From the production process at the plant, silica gel contains F impurities at 21%.

**Figure 1:**
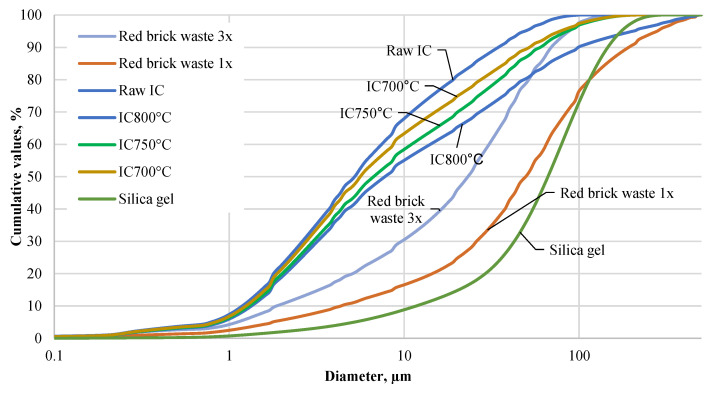
Particle size distribution of AAM precursors.

The particle size distribution of precursors is given in Figure 1. The results indicate that the fineness of freshly ground raw clay gradually decreased after the heat treatment temperature increased from 700 to 800 °C (IC700 °C, IC750 °C, IC800 °C). Raw IC was associated with d_10_ of 1.29 µm, d_50_ of 5.50 µm, and d_90_ of 36.53 µm. Treating red IC at 800 °C particle size increased d_10_ to 1.44 µm, d_50_ to 8.26 µm, and d_90_ to 108.21 µm, respectively. Ground red brick waste with similar grinding parameters as for thermally treated raw IC (red brick waste 1x) showed a much coarser particle size distribution. The particle size of such ground red brick waste was d_10_ of 4.66 µm, d_50_ of 52.65 µm, and d_90_ of 202.20 µm. To reduce red brick waste particle size, additional grinding (three times more energy) was applied with the same grinding parameters (red brick waste 3x). An additional grinding of red brick waste reduced its particle size: d_10_ to 2.07 µm, d_50_ to 24.64 µm, and d_90_ to 74.19 µm. The density of raw IC was 2.76 g/cm^3^, while thermal treatment reduced this value from 2.69 to 2.74 g/cm^3^. The thermal treatment of raw IC reduced specific surface area. The specific surface area of raw ground IC was 4155 cm^2^/g, and the thermal treatment reduced this value from 3925 to 3475 cm^2^/g. A higher temperature was associated with a lower specific surface area (IC800 °C). For ground red brick waste, the specific surface area was 1448 cm^2^/g and with additional grinding, it increased to 2515 cm^2^/g. The specific surface area of silica gel was 716 cm^2^/g and its particle size distribution was accordingly d_10_ 11.71 µm, d_50_ 66.39 µm, and d_90_ 146.12 µm.

**Figure 2:**
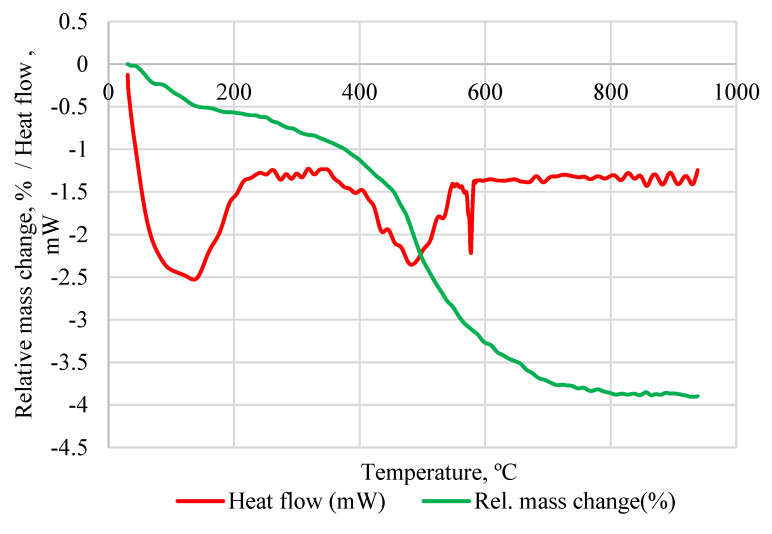
DTA/TG of raw IC.

According to DTA/TG analysis of IC, the total mass change during the analysis was −3.8% (Figure 2). The first mass change peak is associated with free water desorption between 35 and 214 °C and the mass change there was −0.67%. An additional −1.97% mass change was detected between 411 and 546 °C. This is associated with the dehydroxylation of IC which is in a similar interval as previously detected for kaolin clay [5]. The obtained value was significantly lower if compared to the dihydroxylation value for kaolin clay which according to the literature is about 8.5% [19,20]. This could be an indicator that relatively less reactive phases are present in calcined IC. Between 573.4 and 580.9 °C the mass change was −0.14% which is an indication of the allotropic transformation of quartz which also corresponds with the data from the XRD and literature [21].

**Figure 3:**
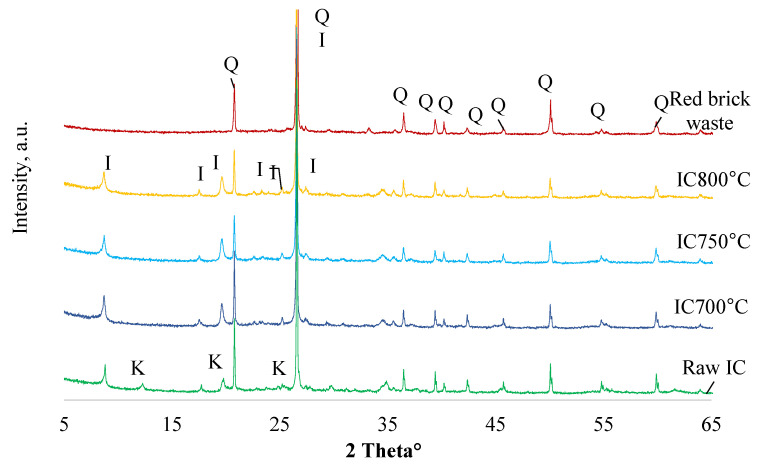
The XRD patterns of raw IC, red brick waste, and calcined red clay at different temperatures. Notes: Q is quartz SiO_2_ (77-1060), I is illite (26-911), K is kaolinite (83-971).

The mineral crystalline phase transformation of raw IC during the heat treatment between 700 and 800 °C (IC700 °C, IC750 °C, IC800 °C) and red brick waste through the XRD characterization is given in Figure 3. The main minerals detected in IC were quartz SiO_2_ (77-1060), illite (26-911), and kaolinite (83-971). Heat-treated IC had lost its kaolinite peak. The illite crystalline phase was not detected for red brick waste precursor. The silica gel contained crystalline aluminum fluoride hydrate and an amorphous region was identified between 20 and 30°, as reported in the previous paper [16].

### 2.2. Mixture Compositions

Two series of AAMs were prepared using calcined IC (700 and 800 °C) and ground red brick waste (Br1x and Br3x) at two milling regimes as precursors (Table 2 and Table 3). The first composition series were prepared with heat-treated IC (700 and 800 °C) or ground red brick waste as precursors. 6 M and 7 M NaOH solution were used for alkali activation [22,23]. Then combination of red brick waste and thermally treated clay was prepared with the replacement ratio of each clay by 25 wt.%. The fineness of the precursor played a role in the content of the alkali activation solution (AAS). Finer heat treated clay needed an extra amount of AAS and the AAS—Calcined IC precursor (AAS/solid) was 0.6. For coarser red brick waste precursor, the AAS ratio to precursor was 0.47. A mix of both clays gradually increased AAS content as calcined IC content increased in the composition. The second series of AAM consisted of red brick waste and 5 wt.% addition of silica gel activated with 6 M or 7 M NaOH solution. AAS/solid ratio was 0.43 (Table 3).

Solid raw materials were homogenized before being gradually poured into the AAS. A handheld electrical two-shaft mixer was used to prepare the samples. The material was stirred until it was a homogeneous paste, then it was cast in a silicone mold measuring 20 × 20 × 20 mm. The molds were then coated with plastic film and placed in an 80 °C drying chamber for 24 h to cure. Early age strength was measured after samples were taken out of molds after 24 h of curing. The remaining samples were divided into two groups: half were cured at ambient room temperature (22 ± 2 °C, 50% RH), and the other half were cured in a wet environment (22 ± 2 °C, 95% RH). Room temperature cured samples were examined at the age of 28 days, while moist cured samples were conditioned and dried at 22 ± 2 °C, 50% RH before being tested at 35 days.

### 2.3. Testing Methods

A CILAS 1090 LD laser scattering particle size distribution analyzer was used to determine specific surface area, density, and particle size distribution in the range from 0.1 to 500 µm for the precursors.

The XRD analysis was performed using a D8 Advance diffractometer (Bruker AXS, Karlsruhe, Germany) operating at a tube voltage of 40 kV and a tube current of 40 mA. The X-ray beam was filtered with a 0.02 mm Ni filter to select the CuKα wavelength. The XRD patterns were identified with references available in the PDF-2 database.

A fluorescence spectrometer S8 Tiger (Bruker AXS, Karlsruhe, Germany) was used to determine the chemical composition of raw IC, milled red brick waste, and silica gel. A scanning electron microscope (SEM) FEI Quanta 200 FEG was used to investigate the microstructure of AAM. The chemical elements of the materials were investigated with the Bruker Quad 5040 energy-dispersive X-ray spectrometer (EDS) detector (123 eV).

The specific surface area was measured using the semi-automatic Blaine instrument (TESTING Bluhm & Feuerherdt GmbH, 1.0290 E) according to EN 196-6:2010.

TG/DTA curves were registered with the Linseis STA PT-1600 thermal analytical instrument up to 1000 °C (with a temperature rise rate of 10 °C/min; the air was used as the oxidative environment; the weight of the sample was (50 ± 5) mg).

Mechanical properties of the AAM samples with dimensions of 20 × 20 × 20 mm were performed on a Zwick Z100 universal testing machine with a testing speed of 0.5 mm/min. The curing and testing conditions were described in Section 2.2.

## 3. Results

**Figure 4:**
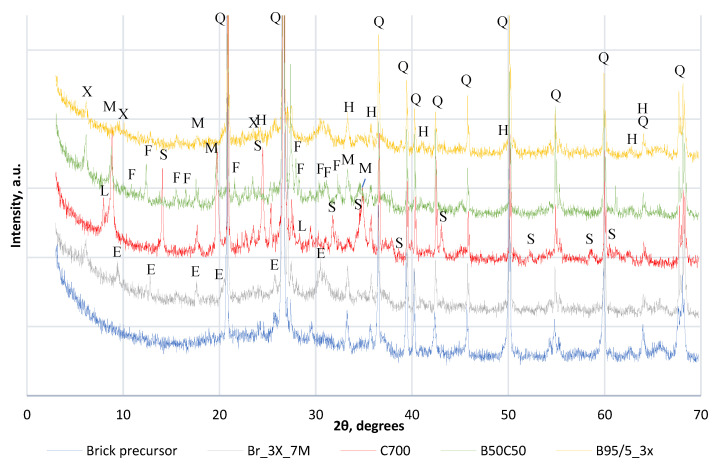
XRD results of AAM. X—faujasite (76-843), Q—quartz (79-1910), H—hematite, syn (13-534), S—hydrosodalite (72-2329), L—montmorillonite (11-303), M—muscovite (7-25), F—philipsite (73-1419), E—herschelite (19-1178).

XRD results of AAM at the age of 35d are given in Figure 4. For all samples, crystalline quartz was detected as a strong peak (79-1910). For the AAM based on calcined IC at 700 °C, montmorillonite, (Al(OH)_2_)_0.33_Al_2_(Si_3.67_A_l0.33_O_10_)(OH)_2_, (11-303) and muscovite, KAl_2_Si_3_AlO_10_(OH)_2_, (7-25) was detected. Hydrosodalite Na_8_Si_6_Al_6_O_24_(OH)_2_(H_2_O)_2_ (72-2329) was also detected. The combination of red brick waste and calcined IC at 700 °C shows the presence of both crystalline peaks detected on AAM with separately activated precursors. Together with muscovite (7-25), philipsite andNa_4_KAl_5_Si_11_O_32_(H_2_O)_10_, (73-1419) were also detected. Different peaks were detected for mixture composition Br3x, where new peaks appeared which are associated with herschelite, NaAlSi_2_O_6_·3H_2_O (19-1178), and X—Faujasite (76-843). For the red brick waste-based sample with silica gel addition (BR95/5), besides muscovite (7-25), and hematite, syn (13-534), zeolitic mineral X—faujasite, (Na_2_Ca )_0.075_(Al_0.3_Si_0.7_)O_2_(H_2_O)_0.22_, (76-843) were detected.

During the curing of the samples, efflorescence was observed in samples with silica gel addition. This white powder was collected from the surface of the samples and tested. XRD analysis of this powder was performed. Results indicate it is an amorphous substance with strong villiaumite, syn, NaF, (36-1455) compound peaks determined. A halo in the region from 20 to 30° 2θ indicates amorphous silica present in this powder.

**Figure 5:**
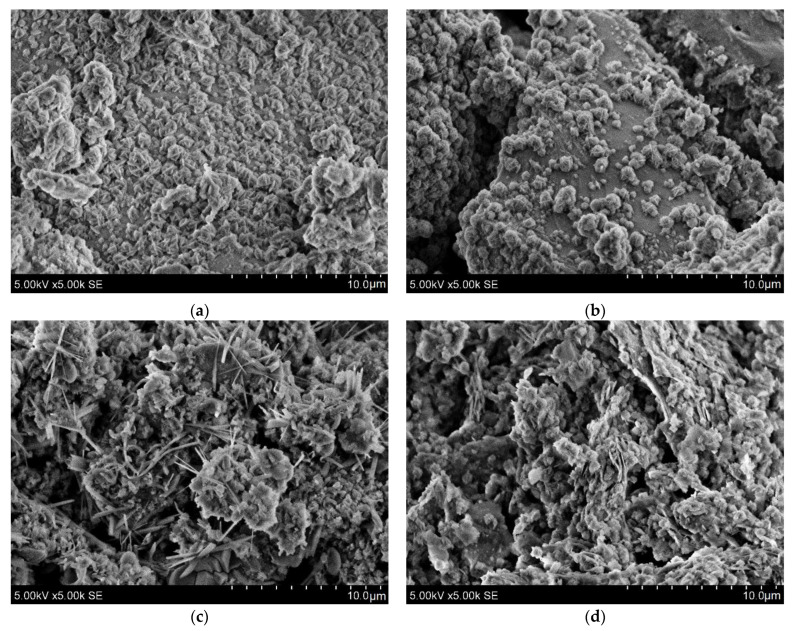
SEM images of AAM prepared from different precursors: (**a**) Br3x; (**b**) B95/5_Br3x; (**c**) B50C50; (**d**) C700.

SEM micrography was used to study the microstructure of four mixtures of different AAM (Figure 5). The energy dispersive spectroscopy (EDS) was performed on SEM images for the chemical characterization of the geopolymerization products. The sample surface is covered with typical hydrosodalite “rose-shaped” individual crystals with a size of around 1.5 μm [24]. The samples based on red brick waste precursor had the highest hydrosodalite crystal coverage. Samples with calcined IC precursor and the red brick waste mixture had different morphology. For composition B50, needle-like and reticular C-S-H phases were embedded within the matrix [25,26].

**Figure 6:**
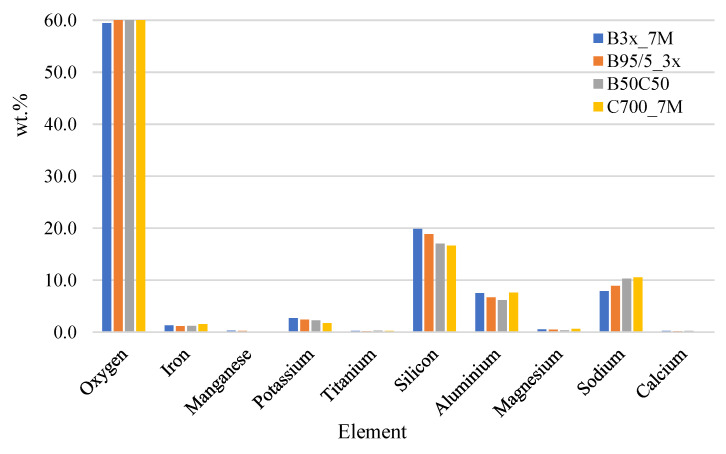
The EDS results of AAM with different compositions.

The EDS test results are shown in Figure 6. The oxygen content of the AAM matrix was from 59 to 61 wt.%, aluminium from 6.2 to 7.6 wt.%, silicon from 16.7 to 19.9 wt.%, and Na from 7.9 to 10.5 wt.%.

**Figure 7:**
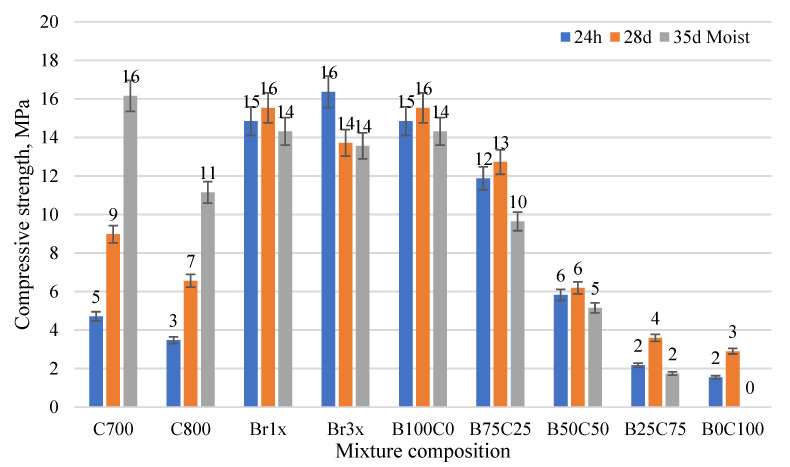
Compressive strength of AAM based on different red clay precursors.

The compressive strength results are given in Figure 7. The compressive strength of calcined IC at 700 °C showed a growing trend during 1, 28, and 35 days of curing. Early compressive strength was 5 MPa and it increased to 16 MPa. IC precursor obtained at higher temperatures (800 °C) showed lower strength gain and strength increased from 3 to 11 MPa. Compressive strength with red brick waste precursor showed rapid strength increase at early age reaching its maximal valuearound 15 to 16 MPa.

**Figure 8:**
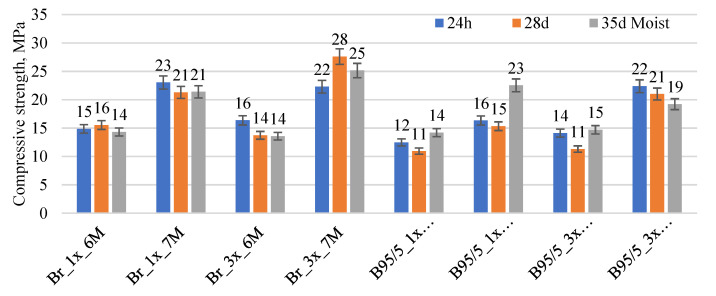
Compressive strength of AAM based on different red brick waste precursors.

The compressive strength results of red brick waste precursor-based AAM are given in Figure 8. The use of higher molarity AAS led to a compressive strength increase for all mixture compositions. Red brick waste precursor which is of a courser nature and activated with 6 M NaOH (6M 1x) showed strength results at around 15–16 MPa at all ages. The extra grinding of red brick waste did not affect strength with 6M NaOH AAS. Compressive strength for 6M 3x was 14–16 MPa. Alkali activation with 7 M NaOH AAS leads to strength increase, for 7M 1x it was 21–23 MPa and for 7M 3x it was 22–28 MPa. The addition of silica gel at 5 wt.% slightly reduced the compressive strength of AAM and it was in the range from 11 to 14 MPa. 7M AAS increased these results from 15 to 23 MPa. A similar tendency was observed from additional ground red brick waste. Compressive strength slightly increased to 11–15 MPa (6M 3x 95/5) and from 19 to 22 MPa for 7 M 3x 95/5.

## 4. Discussion

A geopolymerisation reaction of the precursor was detected as new crystalline peaks were identified after alkali activation of a red brick waste and calcined IC. Minerals such as montmorillonite and muscovite in the composition of AAM samples made with 700 °C treated IC precursor could indicate that polymerization reaction was not completed and these could be remaining from red clay precursors. This also corresponds to the gradual strength increase during the curing period of specimens made with this precursor. Geopolymerisation secondary reaction products such as hydrosodalite were detected which act as an indication for the partial crystallization of sodium aluminate silicate hydrates (N-A-S-H gel) [25,27]. For the mixture composition with a combination of red brick waste and calcined IC at 700 °C, philipsite crystalline phase was detected which could be a further crystallization product of hydrosodalite. The detected needle-like crystals could be referred to the newly crystallized Na-aluminosilicate phase, which could represent zeolite nanocrystals [11]. New zeolite minerals such as herschelite and X—faujasite were detected for AAM based on red brick waste precursors (Br3x). For AAM with silica gel addition, the higher amount of reactive silica initiated the formation of zeolites as fluoride from silica gel may react as a mineralizator. NaF was detected as white efflorescence powder is evidence of an exchange reaction between Al_3_F and NaOH from an alkali activation solution. Villiaumite and gibbsite (Al (OH)₃) can be formed. Similar peaks were observed for AAM with 5% silica gel addition, but the peak intensity was lower as the AAM matrix overwhelmed the quantity of salt in the sample. Such formation of different types of zeolites must be studied in detail to investigate the possibility of forming stable crystalline zeolites with various applications.

SEM results indicated, that the degree of reaction for AAM based on red brick waste precursor is much denser leading to a higher degree of geopolymerisation contributing to a higher compressive strength [28]. EDS results indicate that there is a similar compound content for all mixtures as the same raw IC was used for the preparation of precursors only with different temperature treatment conditions. Compared to metakaolin-based geopolymers which possess O from 41–43%, aluminium from 11.6 to 16.5 wt.%, silicon from 24.5 to 26 wt.% and sodium from 5.2 to 7.4 wt.%, the oxygen and sodium content has increased while the aluminium and silicon content is lower [28]. This is also allocated from the chemical composition of the precursor and activator solution.

The compressive strength results indicate that the red brick waste precursor shows a higher initial strength increase compared to the calcined IC precursor. This could be associated with higher temperature treatment and the increase of reactive phase in clay. Despite the coarser particle size distribution for red brick waste and following lower specific surface area (which could reduce the rate of geopolymerisation during alkali activation), the AAS-solid ratio was lower meaning that a denser structure of AAM was achieved which resulted in higher early compressive strength. Similar tendencies were observed for both red brick waste precursor types ground with different powder fineness or particle sizes milling regimes. A combination of calcined IC precursor and red brick waste precursor leads to a compressive strength decrease. Red brick waste activated with higher molarity NaOH solution showed in general a higher compressive strength and is a favorable option in alkali activation solution selection. Silica gel addition slightly reduced the strength of AAM which corresponds to previously published data [14,16]. Obtained strength results fit well in the traditional AAM strength range giving a reasonable option to replace traditional cementitious binders.

## 5. Conclusions

Heat-treated illite clay (IC) at 700 to 800 °C was used as a precursor for obtaining AAM which corresponds to DTA/TG results, indicating the dehydroxylation of IC at a range from 411 to 546 °C. A comparison to red brick waste precursor from a brick production factory was conducted. The precursor fineness was determined based on grinding parameters, and it was concluded that the grinding of soft raw clay followed by heat treatment provides a finer particle size than the grinding of red brick waste particles (3925 cm^2^/g to 1448 cm^2^/g). Additional grinding of red brick waste allowed us to increase the fineness to 2515 cm^2^/g. A high early strength was obtained with no strength gain during further curing for AAM based on ground red brick waste. Both the 6 M and 7 M NaOH solutions proved to be suitable to produce AAM with strength from 11 to 28 MPa. The coarser particle nature of clay treated at higher temperatures led to lower strength at 35 d from 11 to 16 MPa. The highest strength results were obtained for AAM based on red brick waste. Additionally ground red brick waste with 7 M NaOH alkali activation solution led to 28 MPa strength. An important difference was detected between thermally treated IC and red brick waste precursors—strength gain was more rapid for red brick waste precursor AAM while thermally treated red clay had a lower initial strength and it gradually increased. The addition of silica gel led to a compressive strength decrease and efflorescence was detected with a salt consisting of NaF compounds. The hydrosodalite and zeolite crystals were detected in the structure of AAM based on the red brick waste precursor. Results indicate the difference in characteristics of AAM based on similar source IC precursors, showing the important role of the proper treatment of a precursor before alkali activation. Red brick waste from the brick production process proved to be highly suitable for the production of AAM.

## Figures and Tables

**Table 1:** Elemental composition of silica gel, raw IC, and red brick waste from Lode detected by XRF, mass, %.

Compound	Silica Gel	Raw IC	Red Brick Waste
Al_2_O_3_	5.7	14.5	14.5
SiO_2_	72.2	70.3	68.4
CaO	0.4	0.3	0.6
TiO_2_	-	0.9	1.3
Na_2_O	-	0.1	-
K_2_O	-	4.0	5.7
MgO	-	1.1	1.1
Fe_2_O_3_	0.7	5.4	6.6
F	21	-	-
Others	-	1.4	0.4
LOI, 1000 °C	-	2.0	-
Total	100	100	100

**Table 2:** AAM compositions based on heat-treated IC and ground red brick waste precursors, wt.%.

	Composition	6 M NaOH Solution	Red Brick Waste	Calcined IC	AAS/Solid
1	C700	60	-	100	0.60
2	C800	60	-	100	0.60
3	Br1x	43	100	-	0.43
4	Br3x	43	100	-	0.43
5	B100C0	43	100	-	0.43
6	B75C25	47	25	75	0.47
7	B50C50	52	50	50	0.52
8	B50C50 7 M	43	50	50	0.43
9	B25C75	56	70	25	0.56
10	B0C100	60	-	100	0.60

**Table 3:** AAM with red brick waste precursor with different fineness activated with 6 or 7 M NaOH and with silica gel addition, wt.%.

Composition	AAS	Red Brick Waste 1x	Red Brick Waste 3x	Waste Silica gel	AAS/Solid
Br_1x_6 M	43	100	-	-	0.43
Br_1x_7 M	43	100	-	-	0.43
Br_3x_6 M	43	-	100	-	0.43
Br_3x_7 M	43	-	100	-	0.43
B95/5_1x_6 M	43	95	-	5	0.43
B95/5_1x_7 M	43	95	-	5	0.43
B95/5_3x_6 M	43	-	95	5	0.43
B95/5_3x_7 M	43	-	95	5	0.43

## Data Availability

Not applicable.
